# Actin cytoskeleton remodeling defines a distinct cellular function for adhesion G protein-coupled receptors ADGRL/latrophilins 1, 2 and 3

**DOI:** 10.1242/bio.039826

**Published:** 2019-04-04

**Authors:** Judith S. Cruz-Ortega, Antony A. Boucard

**Affiliations:** Department of Cell Biology, Centro de Investigación y de Estudios Avanzados del Instituto Politécnico Nacional (CINVESTAV-IPN), México City 07360, México

**Keywords:** Cell adhesion molecules, G protein-coupled receptor, Intercellular adhesion, Latrophilin, Actin cytoskeleton, Synapse

## Abstract

Latrophilins represent a subgroup of the adhesion G protein-coupled receptor family, which bind to actin-associated scaffolding proteins. They are expressed in various tissues, suggesting that they might participate in biological processes that are ubiquitous. Here we focus on actin cytoskeleton dynamics to explore the role of latrophilins in mammalian cells. Individual overexpression of each latrophilin isoform comparably increased cell volume while modifying the net profile of F-actin-dependent cell extensions, as evaluated by confocal microscopy analysis. Latrophilin deletion mutants evidenced that direct coupling to the intracellular machinery was a requirement for modulating cell extensions. The association between latrophilins and the actin cytoskeleton was detected by co-immunoprecipitation assays and corroborated with immunocytochemistry analysis. Consistent with the destabilization of F-actin structures, latrophilin isoforms constitutively induced a prominent increase in the activity of actin-depolymerizing factor, cofilin. Intercellular adhesion events stabilized by heterophilic Teneurin-4 trans-interactions disrupted latrophilin colocalization with F-actin and led to an isoform-specific rescue of cell extensions. Thus, we find that the actin cytoskeleton machinery constitutes an important component of constitutive as well as ligand-induced signaling for latrophilins.

This article has an associated First Person interview with the first author of the paper.

## Abstract (precis)

**Summary:** Synapses involve the adhesion function of latrophilins within existing neuronal extensions. We show that latrophilins engage the actin cytoskeleton, both constitutively and upon ligand stimulation, to dictate cell extension patterns.

## INTRODUCTION

Latrophilins (Lphn), members of the adhesion G protein-coupled receptor (aGPCR) family, play a key role as stabilizers of neuronal synapses and in synaptic functions linked to behavior (Anderson et al., 2017; Boucard et al., 2014; Lange et al., 2012; O'Sullivan et al., 2014; Wallis et al., 2012). Their adhesion function at the synapse relies on the presence of two adhesion motifs designated as the lectin and olfactomedin-like domains, known to interact with endogenous ligands such as teneurins (Boucard et al., 2014; Silva et al., 2011). However, the distribution of mammalian latrophilins (Lphn1, 2 and 3) in tissues such as lung, heart, kidney and pancreas hint that roles are not restricted to synaptogenesis, but such evidence is sparse (Ichtchenko et al., 1999; Matsushita et al., 1999; Sugita et al., 1998). Cleavage at an auto-proteolytic site, named the GPS site, generates a C-terminal fragment comprising Lphn seven-transmembrane domains with interconnecting loops and a C-terminal intracellular tail which is able to recruit actin-binding scaffolding proteins (Boucard et al., 2012, 2014; Kreienkamp et al., 2000; O'Sullivan et al., 2012; Silva et al., 2011; Tobaben et al., 2000). However, there is no evidence so far that Lphns can functionally participate in actin reorganization. Additionally, the molecular pathways through which Lphns translate adhesion events into intracellular signals remain elusive.

Actin, an essential component of the eukaryotic cytoskeletal framework, plays an active role in both adhesion and/or migration events (Katsuno et al., 2015; Nobes and Hall, 1999; Pollard and Borisy, 2003). Its globular fraction is readily polymerized into filaments by different factors that help elaborate cell architecture, thereby giving rise to distinct cell dynamics (Katsuno et al., 2015). Filopodia, lamellipodia and blebs are amongst the structures that are stabilized by filamentous actin (F-actin) and support cell phenotypes linked to migration (Mejillano et al., 2004; Small and Celis, 1978).

Thus, we aimed at elucidating how Lphns modulate actin dynamics to support their adhesion function in mammalian cells.

## RESULTS AND DISCUSSION

### Latrophilins regulate cellular and nuclear morphology

**Fig. 1.**
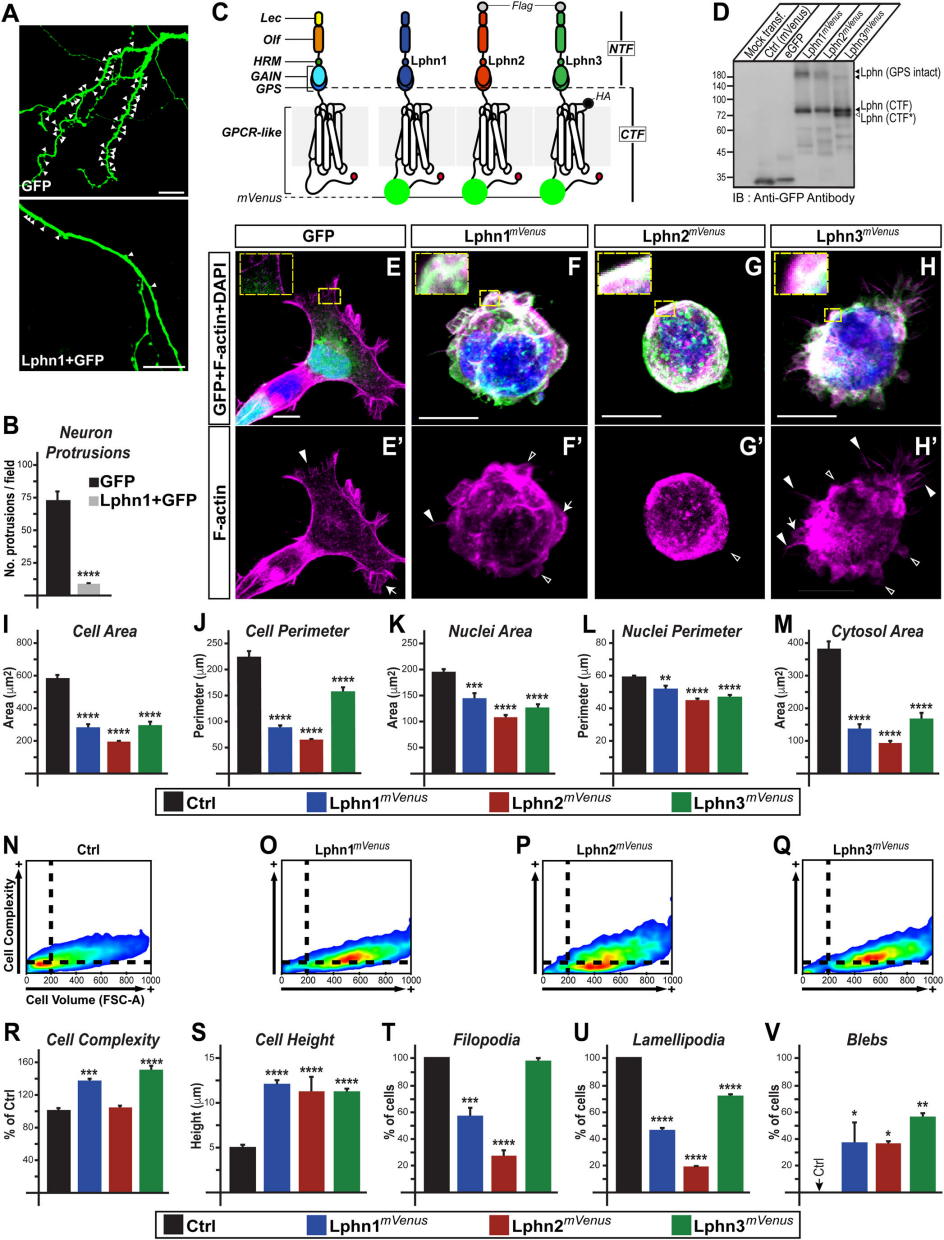
**Latrophilins exert a cell-autonomous effect on cell morphology and the genesis of cell extensions.** (A,B) Transfected hippocampal neurons expressing indicated proteins and quantification of neuronal protrusions; white arrowheads indicate sites of protrusions. (C) Schematic representation of Lphn*^mVenus^* fusion proteins used in this study with HA and Flag epitopes where indicated: lectin (Lec), olfactomedin (Olf), hormone binding (HRM), GPCR auto-proteolysis inducing (GAIN), GPCR proteolysis site (GPS), N-terminal fragment (NTF), C-terminal fragment (CTF) and a PDZ-binding domain represented as the C-terminal red circle. (D) Cell extracts from HEK293T cells expressing the indicated proteins were analyzed by immunoblotting with an anti-GFP antibody. Lphn (CTF) and Lphn (CTF*) represent two fragments resulting from unknown post-translational modifications. (E–H') Confocal microscopy imaging analysis of fixed cells expressing Lphn*^mVenus^* (in green) and stained for nucleus (DAPI, in blue) and F-actin (in magenta). Selected F-actin cell extensions are indicated: filopodia (filled arrowheads), lamellipodia (arrows) and blebs (hollow arrowheads). (I–M) Cell and nuclei dimensions as well as cytosolic area of transfected cells. (N–R) Flow cytometry analysis of cell complexity and volume for transfected cells (*n*=20,000). (S–V) Confocal microscopy analysis of cell height and population of cells displaying either filopodia, lamellipodia or blebs. Scale bars: 10 µm. Data, represented as mean values, were obtained from at least three separate experiments (neuron protrusion assays: *n*=22; confocal microscopy assays: Ctrl *n*=66, Lphn1*^mVenus^ n*=27, Lphn2*^mVenus^ n*=27, Lphn3*^mVenus^ n*=30). Error bars indicate s.e.m. ****P≤0.0001, ***P≤0.001, **P≤0.01, *P≤0.05.

In order to probe the effect of Lphn on cell morphology, we co-transfected hippocampal neurons with green fluorescent protein (GFP) and Lphn1 constructs to monitor neuronal protrusions. Neurons transfected with Lphn1 construct displayed a significant reduction in neuronal protrusions compared to control neurons (Fig. 1A,B). This result was intriguing given that loss-of-function approaches had also revealed a loss of neuronal protrusions in hippocampal neurons (Anderson et al., 2017; Boucard et al., 2014). The high-order complexity of neuronal systems prompted us to adopt a reductionist approach to gain insights into molecular determinants involving the role of Lphns on cell morphology. Going forward, we opted for a cellular system devoid of functional Lphns to further dissect the cell-autonomous effects of Lphn1, Lphn2 and Lphn3 expression (Boucard et al., 2014; Hlubek et al., 2000). Thus, human embryonic kidney 293T (HEK293T) cells expressing each Lphn isoform fused to the fluorescent protein mVenus were stained to visualize both the F-actin cytoskeleton and their nucleus (Fig. 1C–H). Images captured using confocal microscopy revealed that the area and perimeter of Lphn-expressing cells, as well as their nuclei, were significantly smaller than control cells expressing mVenus alone when viewed along the cell/matrix interface axis (Fig. 1I–L). As a consequence of these morphological changes, Lphn-expressing cells suffered a significant shrinking of their cytosolic area (Fig. 1M). We additionally employed flow cytometry analysis of cell singlets to obtain 3D parameters, which revealed a sharp increase in the volume of cells expressing each Lphn isoform (Fig. 1N–Q). The same analysis identified a select increase in cell complexity for Lphn1*^mVenus^*- and Lphn3*^mVenus^*-expressing cells, which could be the result of an altered membrane architecture (Fig. 1R). Consistent with an increased volume observed in flow cytometry assays, microscopy analysis denoted an increase in cell height (Fig. 1S). Taken together, these data reveal that modulation of cell morphology is a converging function of Lphns.

### Latrophilin isoforms modify the net profile of cell extensions

Cellular extensions, which are present in all mammalian cells that navigate their environment, require elements of the actin cytoskeleton for their formation (Rich and Terman, 2018). Based on our observation that Lphn1 affects neuronal protrusion formation and given the reported association between Lphn expression and actin-rich cell specializations such as growth cones, we tested the hypothesis that Lphns modulate the formation of cell extensions supported by actin polymerization (Nozumi et al., 2009; Volynski et al., 2004). Thus, we characterized morphologically identifiable F-actin structures from transfected HEK293T cells. The presence of filopodia, lamellipodia or blebs was documented for isolated cells to enrich for cell-autonomous phenotypes (Fig. 1T–V). Expression of Lphn1*^mVenus^* and Lphn2*^mVenus^* led to a decrease in the number of cells harboring both filopodia and lamellipodia, while the effect of Lphn3*^mVenus^* overexpression was reflected on the decrease in cells displaying lamellipodia (Fig. 1T,U). This pattern suggests an intrinsic inhibition of small Rho GTPases such as cdc42 and Rac, dedicated to the formation of filopodia and lamellipodia, respectively (Nobes and Hall, 1995). On the other hand, blebs that were initially absent from control HEK293T cells appeared in a significant population of Lphn-expressing cells (∼30–50% of cells) (Fig. 1V), thus suggesting that Lphn expression weakens cortical actin, therefore allowing the cytoplasm to exert outward radial forces on membrane patches (Charras et al., 2008; Fackler and Grosse, 2008). These observations denote that although all Lphns modulate actin structures, their function bears an isoform-specific component.

### Uncoupling of Lphn functions on cell dimensions and cell extensions isoforms from the intracellular machinery highlights functions on cell morphology and structures that are both dependent and independent from their GPCR-like region

**Fig. 2.**
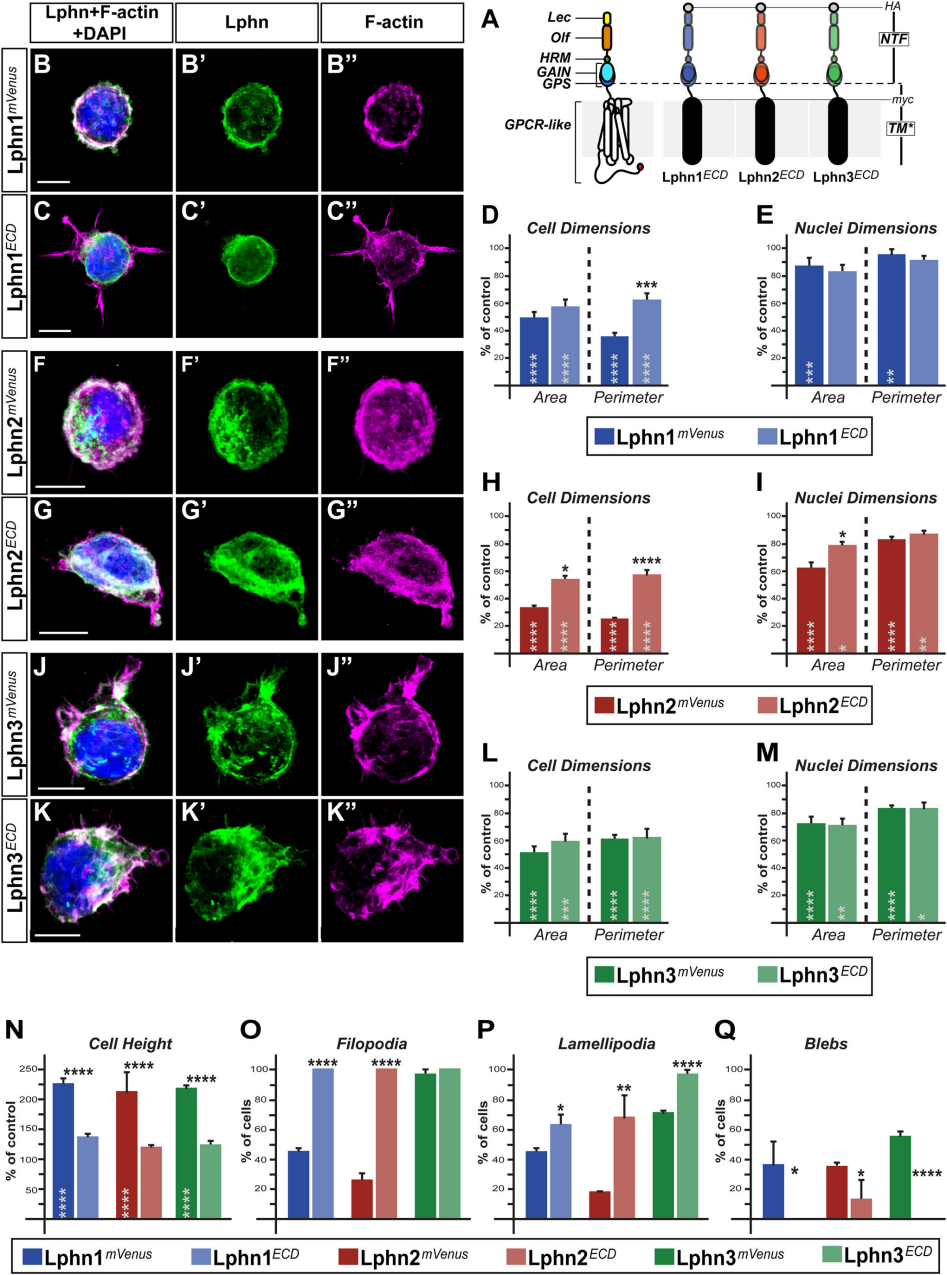
**Uncoupling Lphns from the intracellular machinery distinguishes between NTF- and CTF-dependent actin remodeling functions.** (A) Schematic representation of Lphn^ECD^ proteins containing both HA and myc epitopes followed by the transmembrane domain of platelet-derived growth factor receptor (TM*). Represented domains are: lectin (Lec), olfactomedin (Olf), hormone binding (HRM), GPCR auto-proteolysis inducing (GAIN), GPCR proteolysis site (GPS), seven transmembrane domains and interconnecting loops (GPCR-like), N-terminal fragment (NTF). (B,C,F,G,J,K) HEK293T cells expressing indicated proteins were visualized by confocal microscopy after staining for nuclei (in blue), for F-actin (in magenta) and HA epitope or mVenus fluorescence (in green). (D,E,H,I,L,M,N) Cell and nuclei dimensions as well as cell height values represented as a percentage of mVenus-expressing cells values. (O,P,Q) Percentage of cells harboring filopodia, lamellipodia or blebs. Scale bars: 10 µm. Data, represented as mean values, were obtained from at least three separate experiments (Ctrl *n*=23, Lphn1*^mVenus^ n*=27, Lphn1^ECD^ *n*=29, Lphn2*^mVenus^ n*=27, Lphn2^ECD^ *n*=34, Lphn3*^mVenus^ n*=30, Lphn3^ECD^ *n*=30). Error bars indicate s.e.m. Gray asterisks indicate significance with control values from cells expressing mVenus. ****P≤.0001, ***P≤0.001, **P≤0.01, *P≤0.05.

Latrophilins are linked to the intracellular signaling machinery through the presence of their seven transmembrane domains and interconnecting cytoplasmic regions, the latter displaying a very low inter-isoform sequence homology (Matsushita et al., 1999). Thus, we sought to dissect out the contribution of the GPCR-like region in Lphn-mediated effects on cell size and formation of F-actin structures. For this, only the N-terminal extracellular domains of each Lphn (Lphn^ECD^) were individually expressed as membrane-anchored proteins (Fig. 2A). Cells expressing Lphn^ECD^ isoforms displayed reduced cell and nuclei dimensions compared to control cells (Fig. 2B–M). However, isoform-specific modulation of cell dimensions was detected: Lphn3^ECD^ recapitulated the phenotype of its full-length counterpart, Lphn1^ECD^-expressing cells differed from cells harboring Lphn1*^mVenus^* on cell perimeter measurements only and Lphn2^ECD^ diverged from Lphn2*^mVenus^* in both cell area and perimeter (Fig. 2D,H,L). Nuclei dimensions for Lphn^ECD^-expressing cells kept the same characteristics as for cells expressing their full-length counterparts, except for Lphn2^ECD^ expression, which induced a small but significant difference in nuclei area (Fig. 2E,I,M). These data reveal that molecular signals governing cell and nuclei dimensions are differentially conserved in the N-terminal extracellular domains of either Lphn1, Lphn2 or Lphn3. Surprisingly, the height of cells expressing either of the three Lphn^ECD^ was similar to control cells in contrast to cells expressing their full-length counterparts, suggesting that the GPCR-like region is required to mediate the cell volume phenotype elicited by Lphn expression (Fig. 2N).

We then proceeded to analyze the effect of Lphn^ECD^ overexpression on the formation of cell extensions. Virtually all cells analyzed that expressed Lphn1^ECD^ and Lphn2^ECD^ harbored filopodia, a phenotype that presented a stark contrast to their full-length counterparts' expression (Fig. 2O). Lamellipodia formation for the population of cells expressing Lphn^ECD^ was significantly less affected than for their full-length versions, noting that Lphn3^ECD^ maintained control level values (Fig. 2P). Interestingly, membrane blebbing was inexistent in cells expressing Lphn1^ECD^ and Lphn3^ECD^ while a significantly small population was detected for Lphn2^ECD^ expressing cells (Fig. 2Q). Thus, in contrast to cell and nuclei dimensions, Lphn effects on the formation of cell extensions and cell height clearly converged towards a common requirement for their GPCR-like region. Taken together, these data suggest that the determinants governing cell extensions are at least in part distinct from the ones influencing cell and nuclei dimensions.

### Ligand-mediated intercellular adhesion events modulate Lphn functions on cell dimensions and cell extensions

**Fig. 3.**
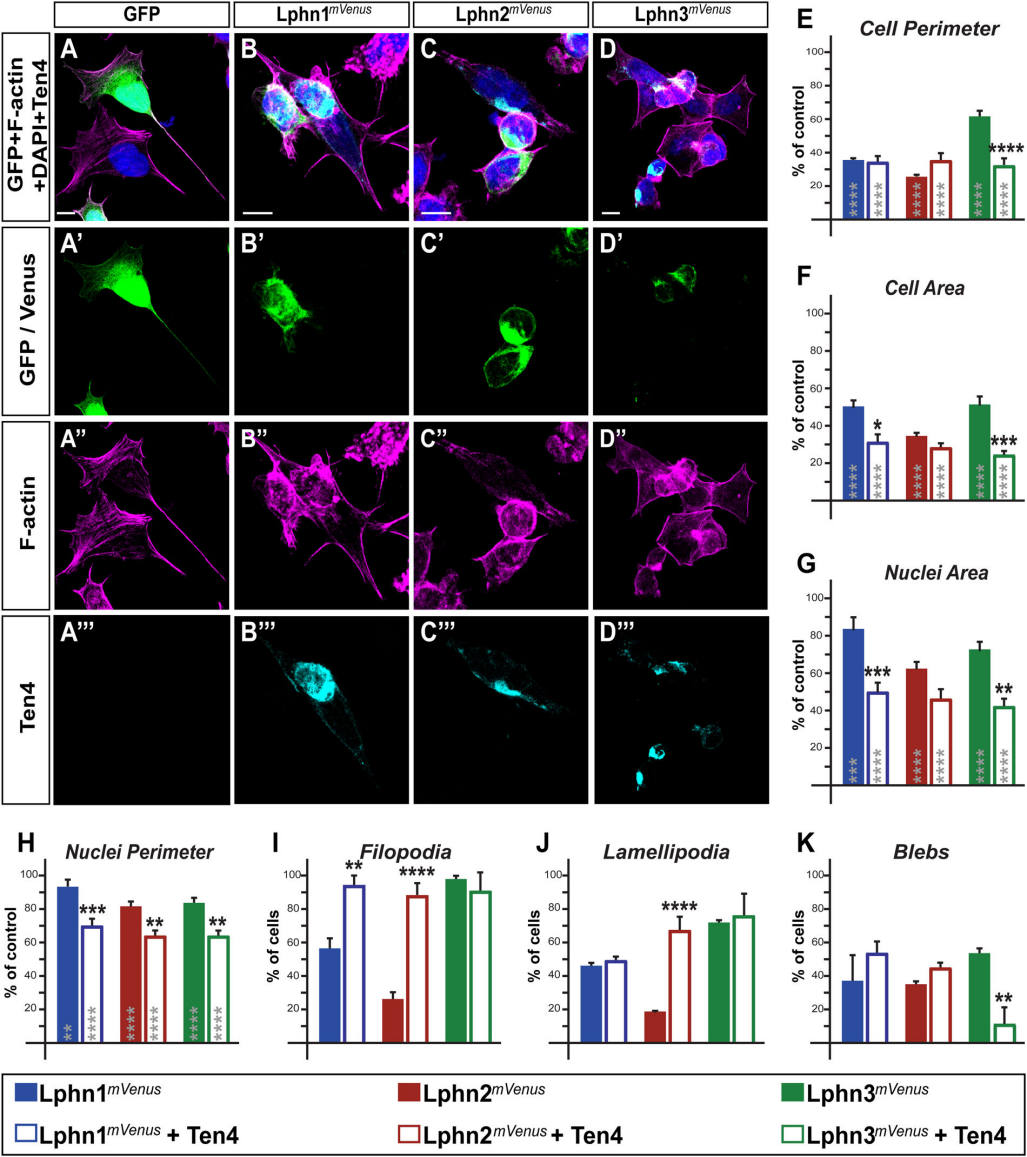
**Intercellular adhesion established between Lphn- and Ten4-expressing cells leads to isoform-specific reversals of cell-autonomous phenotypes.** (A–D‴) Cell aggregates displaying Lphn*^mVenus^* expression (in green) were analyzed using confocal microscopy after staining for nuclei (in blue), F-actin (in magenta) and Teneurin-4 (in cyan). (E–H) Cell and nuclei dimensions of cells expressing the indicated proteins were plotted as a percentage of control mVenus-expressing cells values. (I–K) Percentage of cell population displaying indicated F-actin structures. Scale bars: 10 µm. Data, represented as mean values, were obtained from at least three separate experiments (Ctrl *n*=25, Lphn1*^mVenus^ n*=27, Lphn1*^mVenus^+*Ten4 *n*=15, Lphn2*^mVenus^ n*=27, Lphn2*^mVenus^+*Ten4 *n*=18, Lphn3*^mVenus^ n*=30, Lphn3*^mVenus^+*Ten4 *n*=12). Gray asterisks indicate significance with control values from cells expressing mVenus. Error bars indicate s.e.m. ****P≤0.0001, ***P≤0.001, **P≤0.01, *P≤0.05.

The role of Lphn in neuronal synapse formation involves heterophilic interactions with its ligands teneurins (Ten1–4), which, by binding to the receptors' lectin-like domain, is responsible for mediating cell-cell adhesion (Boucard et al., 2014; Li et al., 2018; O'Sullivan et al., 2012). Thus, we sought to assess changes in morphological parameters of Lphn-expressing cells as a consequence of their contact with cells expressing Teneurin-4 (Fig. 3B–D). Control-transfected cells did not form aggregates and therefore showed a homogenous dispersion while maintaining an elongated morphology (Fig. 3A). Cell and nuclei dimensions for aggregated Lphn-expressing cells remained smaller than control cells but a more profound decrease was observed for cells expressing Lphn1*^mVenus^* and Lphn3*^mVenus^* compared to cell isolates (Fig. 3E–H).

Additional analyses were conducted on aggregated Lphn-expressing cells in order to determine if transcellular adhesion with Ten4 presented alterations in the cell extensions profile (Fig. 3I–K). The loss of filopodia observed in cell isolates for both Lphn1*^mVenus^*-and Lphn2*^mVenus^*-expressing cells was almost completely rescued in cell aggregates with cells expressing Ten4, while the formation of lamellipodia experienced a significant recovery only for Lphn2*^mVenus^*-Ten4 cell aggregates (Fig. 3I–K). Membrane blebbing was detected in all Lphn-Ten4 cell aggregates although the presence of this actin structure was drastically reduced in the combination of Lphn3*^mVenus^*-Ten4 cells compared to isolates of Lphn3*^mVenus^*-expressing cells (Fig. 3K). These data suggest that the cell signaling pathways which incur from teneurin binding to Lphns can provoke an isoform-specific remodeling of the actin-associated structures in HEK293T cells. While the signaling pathway of Lphns towards the actin cytoskeleton remains to be clarified in future studies, our findings extend the similarity between the teneurin system and its ancestral bacterial toxin system. Indeed, modulation of the actin cytoskeleton could represent a conserved evolutionary feature that has led both systems to retain their strategic partnership and targeting of similar cell biological pathways (Lang et al., 2010; Li et al., 2018).

### Latrophilin-actin complex formation and signaling

**Fig. 4.**
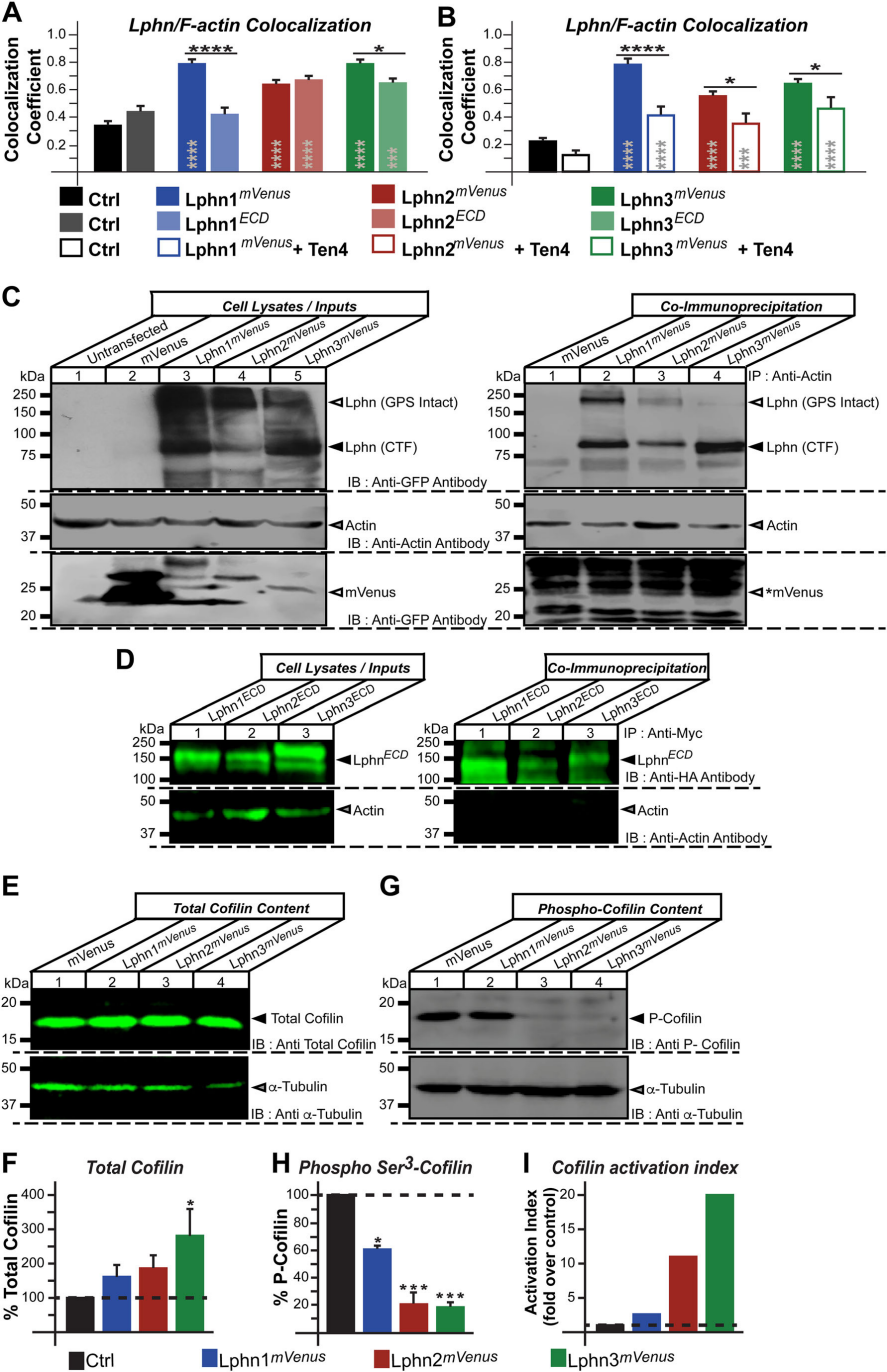
**Latrophilins physically and functionally interact with the actin cytoskeleton of HEK293T cells.** (A,B) Cells expressing the indicated constructs were imaged using confocal microscopy and analyzed for the presence of colocalized fluorescence pixels between Lphn*^mVenus^* and rhodamine-phalloidin stained F-actin in isolates (A) or cell aggregates (B). (C) Immunoprecipitation of actin was conducted on extracts from HEK293T cells expressing the indicated Lphn*^mVenus^* isoforms and subsequently probed for the presence of the receptors or mVenus control using an anti-GFP antibody or immobilized actin using an anti-actin antibody. Arrowheads mark the position of entities that were co-immunoprecipitated with actin (lane 2–4, right panel); asterisk indicates that the control mVenus protein could not be detected in the immobilized fraction despite its high expression (lane 2, left panel versus lane 1, right panel). Note the presence of bands representing Lphn receptors with the GPS intact in each lane corresponding to co-immunoprecipitated receptors (lane 2–4, right panel). (D) Immunoprecipitation using c-MYC antibody-coupled beads was conducted on cell extracts from HEK293T cells expressing the indicated Lphn^ECD^ isoforms and subsequently probed for the presence of actin using an anti-actin antibody. (E,G) Cell extracts from HEK293T cells expressing Lphn*^mVenus^* isoforms were probed with antibodies recognizing total cofilin (E) and phospho-cofilin (G), the inactive form of cofilin, along with an anti-α-tubulin antibody as a loading control. (F,H) Quantification of data obtained in E and G, respectively. (I) Cofilin activation index calculated by dividing total cofilin percentage by phospho-cofilin percentage for each indicated conditions. Data, represented as mean values, were obtained from at least three separate experiments involving 122 cells for microscopy assays in A and 116 cells in B. Gray asterisks indicate significance with control values from cells expressing mVenus. Error bars indicate s.e.m. ****P≤0.0001, ***P≤0.001, **P≤0.01, *P≤0.05.

**Fig. 5.**
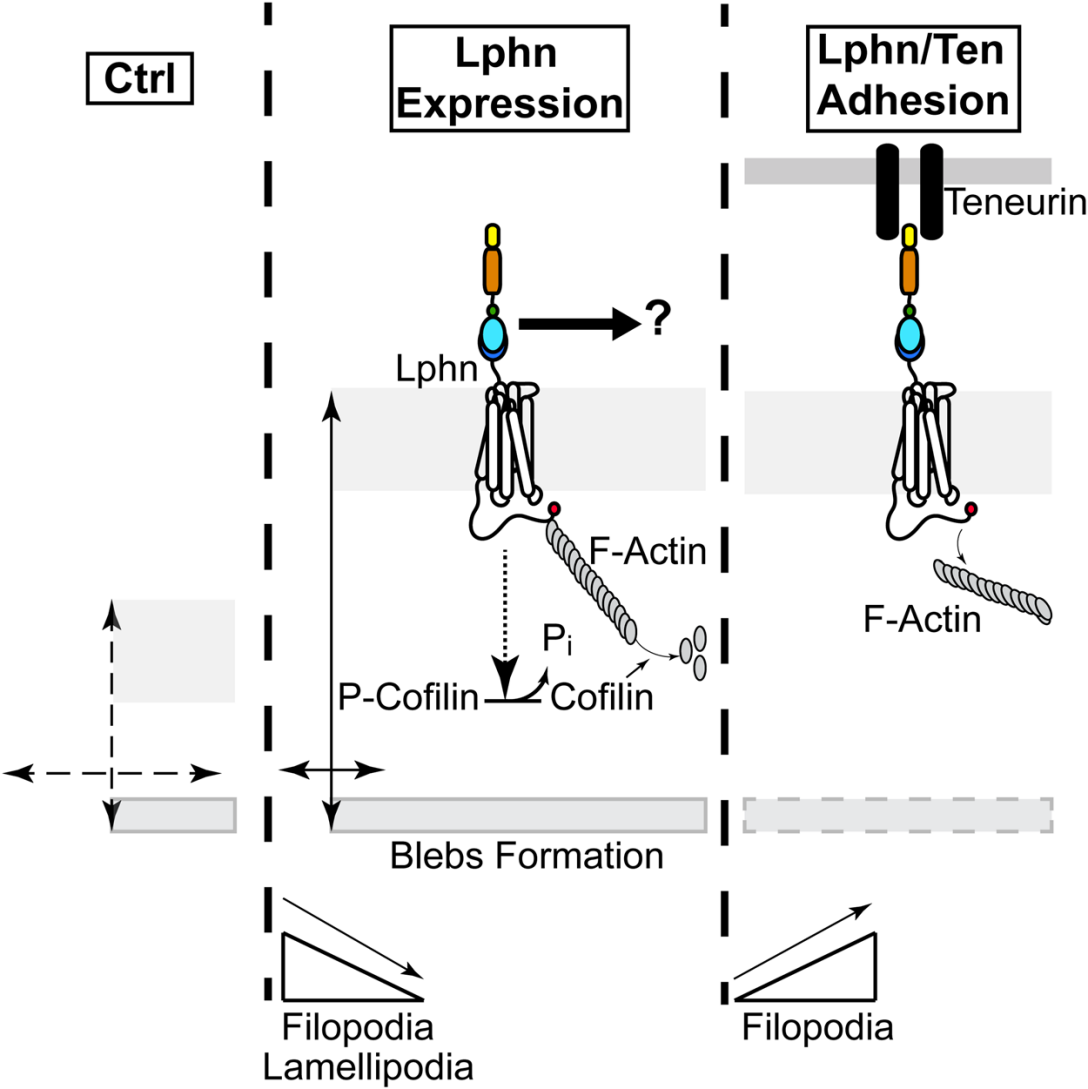
**Schematic representation depicting the effect of Lphns on morphological changes and actin remodeling in HEK293T cells.** Cell membrane expression of Lphn induces an increase in cell volume while decreasing cell-matrix surface contact as compared with control conditions (double-ended arrows). These changes in cell volume are accompanied by a transition in the formation of cell extensions composed of F-actin, such that cells harbor less filopodia and lamellipodia to the benefit of bleb formation, which is normally absent in naive HEK293T cells. The destabilization of F-actin induced by Lphn is corroborated by a constitutive activation of the cofilin pathway. The presence of a physical complex between Lphn and the actin cytoskeleton parallels these effects. The adhesion motif-containing N-terminal domains of Lphn are suspected to interact with a yet unknown molecular pathway to modulate cell and nuclei dimensions. Intercellular adhesion mediated by Lphn and teneurin provoke a net rescue of filopodia while decreasing the Lphn colocalization with the actin cytoskeleton.

Latrophilins’ cytoplasmic tails have been shown to recruit actin-associated scaffold proteins from diverse gene families (Kreienkamp et al., 2000; Tobaben et al., 2000). Thus, we sought to characterize the physical and functional interaction between Lphn isoforms and actin cytoskeleton components in cells. We first evaluated the degree of colocalization between endogenous F-actin and either Lphn*^mVenus^* or Lphn lacking intracellular domains (Lphn^ECD^) expressed in HEK293T cells. Confocal images from single scanning sections were analyzed using the Pearson coefficient to represent the extent of colocalization. The high degree of F-actin colocalization detected for all Lphn isoforms was drastically decreased for cells expressing Lphn1^ECD^ and slightly for Lphn3^ECD^ but not Lphn2^ECD^ (Fig. 4A). Interestingly Lphn2^ECD^ and Lphn3^ECD^ conserved a significant degree of colocalization with F-actin suggesting that their recruitment to the actin cytoskeleton involves their N-terminal domain through a yet unknown mechanism in HEK293T cells (Fig. 4A). This differential pattern of loss in F-actin colocalization partly correlated with a differential effect on F-actin structures since, as previously shown in Fig. 2, F-actin-dependent structures such as filopodia, lamellipodia and blebs approached control values when the GPCR-like region was omitted for all three isoforms. These data point to an isoform-specific role of the N-terminal domain for recruitment to the actin cytoskeleton whereas the GPCR-like region provides the determinants that support Lphn signaling mechanisms which regulate the architecture of cell extensions. Guided by our observation that intercellular adhesion mediated by Lphn-Ten4 interaction induced the recovery of cell extensions, we sought to investigate the level of colocalization between F-actin and Lphn in aggregated cells (Fig. 4B). A significant decrease in colocalization between F-actin and all Lphn isoforms was detected in aggregated cells compared to cell isolates, suggesting that Teneurin-4 stimulates the formation of specific cell extensions (Fig. 3) by inducing a dissociation between latrophilins and F-actin (schematized in Fig. 5).

The apparent colocalization of Lphn proteins with F-actin prompted us to verify if these receptors could form a physical complex with actin in HEK293T cells. Actin immunoprecipitation conducted with cell extracts from Lphn*^mVenus^*-transfected cells led to the concomitant recruitment of an anti-GFP immunoreactive ∼85 kDa band corresponding to the CTF of all three isoforms of Lphn*^mVenus^* proteins but not of control mVenus protein (Fig. 4C). It is noteworthy that a ∼200 kDa band representing the GPS-intact Lphns was detected in these actin-immunoprecipitated cell extracts, indicating that GPS site cleavage does not in itself constitute an absolute requirement for actin recruitment to these receptors (Fig. 4C). In the same scheme of thoughts, we aimed at determining if the presence of Lphn GPCR-like region acted as a stabilizer of actin complex formation. For this, lysates from cells expressing myc epitope-tagged Lphn^ECD^ proteins were immunoprecipitated using anti-myc antibody coupled to protein G agarose beads and the immobilized fractions were probed using anti-actin antibody. In concordance with confocal microscopy analyses, no complex formation between the actin cytoskeleton and Lphn1^ECD^ was evident from these co-immunoprecipitation assays, as these eluates did not reveal detectable levels of actin (Fig. 4D). Similar results were obtained for eluates from Lphn2^ECD^ and Lphn3^ECD^ expression in HEK293T cells, an observation that contrasts with their preferential colocalization with F-actin in confocal microscopy analyses, but which might be explained by the loss of low-affinity complexes during the conduct of high stringency co-immunoprecipitation assays (Fig. 4D). These findings support earlier evidence describing the ability of these receptors to recruit actin-associated scaffold proteins through their GPCR-like region (Kreienkamp et al., 2000; Tobaben et al., 2000).

The pattern of cell extensions in Lphn-expressing cells is reminiscent of an active role elicited by signaling pathways aimed at destabilizing F-actin. The cofilin activation pathway, often called the actin depolymerizing factor pathway, constitutes a major signaling determinant that is involved in reorganizing the actin cytoskeleton. Dephosphorylation of cofilin allows it to bind to both barbed ends and pointed ends of actin filaments resulting in the severing of this cytoskeletal structure (Tanaka et al., 2018; Wioland et al., 2017). Thus, we hypothesized that activation of cofilin could be triggered as part of Lphn signaling pathway sustaining actin cytoskeleton remodeling. Giving that the net activation of cofilin depends on the ratio between phosphorylated and total fractions, we sought to detect both fractions in lysates from Lphn-transfected cells. Evaluation of total cofilin levels indicated that although Lphn1*^mVenus^* and Lphn2*^mVenus^* expression induced a slight increase in cofilin content, only lysates from Lphn3*^mVenus^*-expressing cells reached significance (Fig. 4E,F). In contrast, a drastic dephosphorylation of cofilin was observed in all cell extracts originating from Lphn-transfected HEK293T cells when probed using an anti-phospho-cofilin antibody (Fig. 4G,H). The resulting net content of activated cofilin in Lphn-transfected cells ranged from ∼2–20 times that of control cells (Fig. 4I). These data suggest that Lphns possess a constitutive activity which contributes to destabilizing actin filaments by activating the cofilin pathway (schematized in Fig. 5).

### Actin destabilization: a poorly described GPCR-mediated event

While the involvement of GPCRs in stabilizing actin structures has been widely described, their role in controlling actin depolymerization remains elusive. To date, a pathway involving the stimulation of the GPCR fMLP and leading to the activation of slingshot2, a phosphatase of cofilin, has been shown to require the functional targeting of the phosphatases/kinases set PLCbg/PKCb/PKD to sustain neutrophils chemotaxis (Xu et al., 2015). An alternative model evidencing the modulation of actin polymerization depicts a mechanism in which local translocation and sequestration of cofilin induces the formation of membrane protrusions in different cell types through the participation of the scaffolding function of β-arrestin, an intracellular GPCR-interacting protein involved in their desensitization (Pontrello et al., 2012; Zoudilova et al., 2007). This bipartite model involves the β-arrestin1-mediated scaffolding of cofilin with its inactivating lim domain kinase (LIMK) and the interaction of β-arrestin2 with cofilin and its activating phosphatase SSH (Xiao et al., 2010; Zoudilova et al., 2007, 2010). It is still not clear whether Lphn functionally interacts with β-arrestin to spatially sequester cofilin, but such a mechanism remains an attractive hypothesis that will be tested in future studies.

## MATERIALS AND METHODS

### Plasmids

#### Full-length Lphn expression vectors fused to mVenus

*Lphn1^mVenus^* expression construct was described previously (Boucard et al., 2012) and engineered by introducing the sequence for mVenus into a Mfe I site resulting from its introduction into pCMV-Lphn1 between Ala^1294^ and Lys^1295^ (amino acid numbering from peptide signal Met^1^: rat Lphn1 isoform lacking both SSA and SSB known as CL1AA, accession no.: AF081144) using PCR mutagenesis, therefore adding an Asn-Cys as a linker on both sides of mVenus sequence. *Lphn3^mVenus^* expression construct was generated by introducing mVenus into a Bgl II site resulting from PCR mutagenesis of pCMV-Lphn3*^HA,Flag^* (Araç et al., 2012) at Arg^1277^-Ser^1278^ and thus comprised both HA and Flag epitopes. The following oligonucleotides were used to introduce BglII site: Forward 5′- CAATCATGAAAGATCTAGTGAGCA-3′, Reverse 5′- TGCTCACTAGATCTTTCATGATTG-3′. The following oligonucleotides were used to amplify mVenus flanked by BglII sites: Forward 5′- TATAAGATCTATGGTGAGCAAGGGCGAG −3′, Reverse 5′-TATAAGATCTCTTGTACAGCTCGTCCATG −3′. *Lphn2^mVenus^* expression construct was a generous gift from Thomas Südhof (Stanford University) and consisted of the insertion of mVenus at a C-terminal Pml I unique site of pCAAG-Lphn2^Flag^ comprising an N-terminal Flag epitope.

mVenus expression vector was generated by introducing its full coding sequence into BglII-BamHI sites of pCMV5 using PCR amplification with the following oligonucleotides: Forward 5′-TATAAGATCTATGGTGAGCAAGGGCGAG-3′, Reverse 5′- TATAGGATCCCTTGTACAGCTCGTCCATG.

#### Latrophilin N-terminal extracellular domain expression vectors

*Lphn2^ECD^* and *Lphn3^ECD^* expressing vectors (pDisplay-Lphn2 and pDisplay-Lphn3) were generated by amplifying the regions corresponding to Gly^25^-Lys^848^ and Phe^20^-Asp^874^ respectively of Lphn2 and Lphn3, of which fragments were inserted into the SacII-SalI and XmaI-SacII sites of pDisplay vector respectively. Both sequences were sandwiched between HA and c-Myc epitopes. The following oligonucleotides were used to amplify Lphn2 and Lphn3 extracellular domains: *Lphn2* Forward 5′- TCCCCGCGGATTCAGCAGAGCAGCCTTGC −3′, Reverse 5′-ACGCGTCGACTTTGTGGACGCCATCTTTG −3′; *Lphn3* Forward 5′- TCCCCCGGGTTCAGCCGTGCCCCAATTC −3′, Reverse 5′-TCCCCGCGGATCGTGGACGGCATCGCTG −3′. *Lphn1^ECD^* expression vector (pDisplay-CL1) has been described previously (Boucard et al., 2012).

#### Teneurin

Teneurin-4 expression vector encoding Ten4^HA^ was as previously described (Boucard et al., 2014).

### Antibodies and reagents

Mouse monoclonal anti-hemagglutinin (HA) antibody was from BioLegend (Clone 16B12, Cat. No. 901513). Polyclonal GFP antibody was from Novus Biologicals (Cat. No. NB600-308). Rabbit polyclonal anti-phospho cofilin antibody was from Cell Signaling Technology (Cat. No. C02-3311S). Mouse monoclonal anti-cofilin antibody was from Santa Cruz Biotechnology (Cat. No. sc-376476). Mouse monoclonal anti-actin antibody was a gift from Dr José Manuel Hernández (CINVESTAV-IPN). Alexa-Fluor 633 secondary antibody was from Invitrogen (Cat. No. A21052). 4′,6-diamidino-2-phenylindole (DAPI) and Phalloidin-Rhodamine reagents were from Thermo Fisher Scientific (Cat. No. D1306 and R415, respectively). Mouse monoclonals anti-α-tubulin and anti-c-MYC antibodies were from Developmental Studies Hybridoma Bank (clone 12G10 and 9E10, respectively). Antibodies were used at a 1:1000 ratio unless otherwise specified. Unless otherwise stated, chemical reagents were from Merck-Sigma-Aldrich.

### Primary hippocampal neuronal culture, transfections and morphology analysis

Hippocampal neurons were dissected from DIV0 mice pups (C57BL/6J) in compliance with NOM-062-ZOO-1999 (Mexican official norms) and approved by the Internal Committee for the Care of Laboratory Animals (CICUAL). Neurons were maintained in 24-well plates containing poly-L-lysine-coated (Sigma-Aldrich, Cat. No. P2636) coverslips in plating media, which was replaced the next day by neuronal culture media containing 1% B-27 (Gibco, Cat. No. 17504-010), 2.5% fetal bovine serum (GE, Hyclone Cat. No. SH30071.03) and 25 nM L-glutamine (Gibco Cat. No. 12403-010). Four days later, neuronal cultures were supplemented with 2 mM cytosine arabinoside (Ara-C, Sigma-Aldrich, Cat. No. C6645) and finally completed to 4 mM Ara-C at day 8. At DIV10, neurons were transfected with indicated plasmids using the calcium phosphate method (Xia et al., 1996). Subsequently, neurons were fixed at DIV14 using 4% paraformaldehyde [in PBS for 10 min on ice and blocked with PBS containing 3% BSA (Sigma-Aldrich, Cat. No. A-4503) and 0.1% TritonX-100 (Sigma-Aldrich, Cat. No. X-100)] for 30 min at room temperature. Cells were finally washed again three times with blocking solution and once with water before mounting on microscopy slides using commercial mounting media (Invitrogen, Cat. No. P36966). Slides were then analyzed by acquiring images with a confocal microscope Leica TCS2. The same confocal acquisition settings were applied to all samples of the experiment. Fields of view corresponding to 2× digital zoom through a 63× objective were obtained. Collected z-section images were analyzed blindly using Leica confocal software. For each independent experiment, primary dendrites of pyramidal neurons, defined as dendrites emanating directly from the soma, as well as secondary dendrites were imaged and manually quantified for the presence of protrusions.

### HEK293T cell culture and transfections

For immunocytochemistry experiments, HEK293T cells (authenticated from ATCC) were cultured in 12-well plates containing coverslips that were previously coated with conditioned media from naïve cells, until they reached 50–60% confluency. The cells were then transfected using 1.4 µg of respective cDNA constructs and 4.2 µg of Poliethylenimine (PEI, Polysciences, Cat. No. 23966-1) to achieve a 1:3 ratio of DNA:PEI. After 16 h, the transfection media was replaced by DMEM (Corning, Cat. No. 50-003-PCR) containing 10% FBS (Biowest, Cat. No. S1810-500), 1X Glutamax (Invitrogen, Cat. No. 35050061) and 1000 U ml^−1^ Penicillin/Streptomycin (In Vitro, Cat. No. A-02) and the cells were allowed to grow for another 32 h before being processed for immunocytochemistry experiments. For expression assays using flow cytometry, co-immunoprecipitation and immunoblotting, HEK293T cells were cultured in six-well plates until they reached 80–90% confluency and then transfected using 4 µg of respective cDNA constructs and 12 µg of PEI. The transfection media was removed after 16 h and the cells were grown for an additional 32 h before being harvested for analysis. Tests for the monitoring of mycoplasma were routinely performed every 6 months using MycoFluor Mycoplasma Detection kit (Invitrogen, Cat. No. M-7006).

### Flow cytometry

Adherent HEK293T cells were individually transfected with plasmids indicated in the figures. After 48 h, the cells were resuspended using 1 mM EGTA in PBS and were incubated for 5 min at room temperature in the presence propidium iodide as a marker for cell viability. Propidium iodide-treated cells were analyzed by flow cytometry using BD LSRFortessa™ equipment (BD Biosciences). An arbitrary number of 20,000 events (*n*=20,000) was sampled in each population of transfected cells to allow for a representative distribution. Cells were analyzed for their expression of mVenus by detecting the fluorescence emitted at a wavelength of 530 nm in the fluorescein-5-Isothiocyanate channel and their level of incorporation of propidium iodide that resulted from an emission at a wavelength of 710 nm in the PerCP channel. Data points corresponding to single cells were selected and the analysis was restricted to cells that fit both criteria of being transfected and viable.

### Cell adhesion assays

Cell-adhesion assays were performed with HEK293T cells as described with slight modifications (Boucard et al., 2014). Briefly, HEK293T cells were individually transfected with the expression vectors indicated in the figures. After 48 h, the cells were detached using 1 mM EGTA in PBS, mixed and incubated under gentle agitation at room temperature in DMEM containing 10% FBS, 50 mM HEPES-NaOH pH 7.4, 10 mM CaCl_2_ and 10 mM MgCl_2_. After 120 min, cell aggregates were plated on coverslips previously coated with conditioned media from HEK293T cultures and incubated for an additional 3 h to allow for sufficient adhesion to occur. Cells were washed with PBS and fixed using 4% paraformaldehyde before being processed for immunocytochemistry experiments.

### Immunocytochemistry

Cells transfected with constructs indicated in figures were washed once with PBS and fixed with 4% paraformaldehyde for 15 min on ice. Fixed cells were permeabilized following an incubation at room temperature for 7 min in 0.1% TritonX-100 and washed with cold PBS. Cells processed as part of the adhesion assays between Lphn*^mVenus^* and Teneurin4^HA^ were incubated at room temperature for 2 h with blocking solution containing 3% BSA in PBS and washed with cold PBS three times. Cells were then incubated during 90 min at room temperature with mouse anti-HA antibody (1:200 ratio) followed by 2 h at room temperature with anti-mouse Alexa Fluor 633 fluorescent antibody (Invitrogen, 1:200 ratio) For all other staining assays, cells were incubated for 60 min at room temperature in the dark with phalloidin-rhodamine solution (1:200 ratio) diluted in PBS containing 1% BSA. Finally, cells were incubated for 5 min at room temperature with 300 nM DAPI solution in PBS buffer for nuclei staining, washed three times with cold PBS and the preparations were allowed to dry before mounting on slides using mounting medium. Slides were then analyzed by confocal microscopy.

### Image acquisition and image analysis

Images were acquired using a confocal microscope Leica SP8. The same confocal acquisition settings were applied to all samples of the experiment. Collected z-stack images were analyzed blindly using Leica confocal software. Maximal projection images served as the analysis material for monitoring cell and nuclei dimension parameters, quantification of F-actin structures and for extracting colocalization Pearson coefficient between F-actin and Lphn*^mVenus^* with the exception of cell aggregation samples for which single scanning sections were used instead, in order to avoid distortion caused by amalgams of cells. Data for cell and nuclei dimensions were obtained manually by using the polygonal tool from Leica software (Leica LAS AF Lite 3.3.10134.0). Filopodia, lamellipodia and blebs were manually identified based on the following morphological criteria described in the literature (Hernández-Vásquez et al., 2017; Laser-Azogui et al., 2014): Filopodia, rod-like protrusions originating from the cell membrane and filled with cortical F-actin; lamellipodia, sheet-like protrusions with a base measuring more than 6 um and which must contain cortical F-actin at its periphery; blebs, round-like protrusions with a base measuring less than 2 um and which may or may not contain cortical F-actin at its border. Cell height was determined using all sections collected for single cells. Pearson coefficient analysis was conducted on the following population sample, Fig. 4A: Ctrl *n*=29, Lphn1*^mVenus^ n*=8, Lphn1^ECD^ *n*=16, Lphn2*^mVenus^ n*=12, Lphn2^ECD^ *n*=20, Lphn3*^mVenus^ n*=12, Lphn3^ECD^ *n*=25; Fig. 4B: Ctrl *n*=24, Lphn1*^mVenus^ n*=8, Lphn1*^mVenus^+*Ten4 *n*=14, Lphn2*^mVenus^ n*=12, Lphn2*^mVenus^+*Ten4 *n*=11, Lphn3*^mVenus^ n*=24, Lphn3*^mVenus^+*Ten4 *n*=7.

### Co-immunoprecipitation assays

Cells transfected with the indicated Lphn*^mVenus^* or mVenus constructs were grown for 48 h and lysed in immunoprecipitation buffer at pH 7.4 containing 25 mM Tris, 150 mM NaCl, 1 mM EDTA, 1% TitronX-100 and 5% glycerol. Cell lysates were cleared of insoluble debris by centrifugation at 13,000×***g*** for 15 min. Cleared lysates were incubated overnight at 4°C with protein G-sepharose beads previously coupled to mouse anti-actin antibody or to anti-c-MYC antibody. Non-specific binding to the beads was reduced by performing multiple washing steps with IP buffer and actin- or Lphn^ECD^-immobilized proteins were solubilized using sample buffer. Solubilized samples were loaded onto 10% SDS-PAGE gels. Gels were transferred onto nitrocellulose membranes and processed using standard procedures. Immunoblotting of the membranes was performed using anti-GFP antibody to detect Lphn-Venus or mVenus proteins, anti-actin antibody or anti-c-MYC antibody to detect Lphn^ECD^.

### SDS-PAGE samples preparation and immunoblotting procedures

HEK293T cells were transfected with constructs indicated in figures. 48 h post-transfection, cells were washed in PBS, stored at −70°C for 30 min and thawed at 37°C for 30 s. Cells were scrapped in cold PBS containing 0.1% BSA and centrifuged to isolate insoluble material comprising cell membranes. Cell pellets were solubilized by adding sample buffer, heated and loaded onto 8% or 12% SDS-PAGE gels depending on whether mVenus or total cofilin/phospho-cofilin were to be assayed respectively. After electrophoresis, gels were transferred onto nitrocellulose membranes (Bio-Rad Cat. No. 1620115) and processed using standard procedures.

### Statistics

Data are expressed as means±s.e.m. and represent the results of at least three independent experiments. Statistical significance was determined by Student's *t*-test for neuronal experiments or one-way ANOVA for the remaining experiments using GraphPad Prism software 6.0.
